# Vomica and Respiratory Distress: A Complicated Lung Hydatid Cyst in a Child

**DOI:** 10.1155/crpe/5283544

**Published:** 2026-09-01

**Authors:** Riccardo Guanà, Antonella Peduto, Maria Grazia Sacco Casamassima, Marco Denina, Marco Petraz, Cristina Giuliano, Giuseppe Farruggio, Lucia Gerbaudo, Fabrizio Gennari

**Affiliations:** ^1^ Pediatric and Neonatal Surgery Unit, Regina Margherita Children Hospital, Turin, Italy; ^2^ Pediatric Unit, S. Croce Carle Hospital, Cuneo, Italy; ^3^ Pediatric Surgery Unit, Children’s Mercy Hospital, Kansas, Missouri, USA, childrensmercy.org; ^4^ Department of Pediatric Science and Public Health, Turin University, Turin, Italy, unito.it; ^5^ Pediatric Radiology Unit, Regina Margherita Children Hospital, Turin, Italy

## Abstract

Hydatid cyst, caused by *Echinococcus* infection, is the most common parasitic lung infection in children. Giant cysts are generally the result of a late diagnosis and are associated with a high risk of spontaneous rupture and life‐threatening complications, including anaphylactic shock. We present the complex management of a large, complicated hydatid cyst of the lung in an 11‐year‐old boy in a nonendemic area. This case highlighted the specific work‐up and surgical treatment of lung cysts as well as the differential diagnosis to consider when treating pulmonary masses or pneumonia.

## 1. Introduction

Hydatid cysts are caused by *Echinococcus* infection, a zoonotic disease endemic to many rural regions of East Asia, Eastern Africa, and South America, where it is associated with animal husbandry, but rare in Western Europe and North America [1].

Orally ingested eggs pass through the mucosa of the intestinal tract and enter the portal venous system. Later, they transform into a larval form in the terminal organ where the eggs are held. Hydatid cysts can occur in any organ, including the liver, lung, spleen, kidney, and brain.

While liver involvement is more common in adults, lung involvement is encountered more commonly in children [2–5]. Pulmonary hydatid cysts in children grow about 1–3 cm per year and can remain asymptomatic for a long time, often being diagnosed incidentally. Hydatid cysts grow faster in the lungs than in the liver because the lungs’ greater elasticity allows them to reach larger sizes. In addition, negative intrathoracic pressure may further promote the expansion of pulmonary cysts, thereby increasing their propensity to rupture [6].

These symptoms are usually caused by the cyst’s mass effect or by its rupture [7].

One distinctive symptom is the vomica, that is, the profuse expectoration of a salty liquid and membrane fragments, which occurs when the cyst ruptures within the bronchial tree.

The diagnosis of hydatid cysts is based on a combination of history, imaging, and serology (Table 1).

**TABLE 1:** Summarizing features of lung echinococcosis.

Initial assessment	Diagnostic imaging	Laboratory tests and serology	Differential diagnosis
History and epidemiology: • Contact with animals (dogs and sheep) • Origin from endemic areas	Chest X‐ray: • Uncomplicated cysts appear as well‐defined round or oval opacities • Ruptured cyst includes the “camel sign” or the “water lily sign”	Serology (ELISA and IHA): lower sensitivity (approximately 50%–60%) for pulmonary cysts compared to hepatic ones	• Pulmonary tuberculosis • Lung abscess • Congenital lung malformations
Clinical presentation: • Cough • Chest pain • Dyspnoea • The sudden onset of vomica is pathognomonic for cyst rupture	Computed tomography: considered the gold standard for determining size, number, and exact location and signs of impending or actual rupture	Confirmatory tests: positive screening results should be confirmed by immunoblot to detect specific antibodies (IgG)	
	Abdominal ultrasound: always indicated to rule out concomitant liver involvement	Blood tests: eosinophilia is present in only 15%–25% of cases	

Chest X‐ray is usually the first examination and shows well‐defined, round opacities; computed tomography (CT) is essential for precisely defining the number, location, and condition (intact or ruptured) of cysts; abdominal ultrasound is routinely performed to rule out concomitant liver involvement; serology (ELISA), even if negative, does not rule out the disease.

However, in nonendemic areas, complicated cysts may pose challenges in differentiating them from other pulmonary lesions, such as lung abscesses, congenital airway malformations, fungal cysts, tuberculosis, or tumors.

The treatment of pulmonary hydatid cysts is surgical removal.

Parenchyma‐sparing surgical approaches, such as enucleation, cystotomy and capitonnage, or pericystectomy, are the preferred surgical methods. However, wedge resection and lobectomy are needed in selected cases, when parenchymal damage is severe or in complications such as bronchiectasis, chronic abscess, or severe bleeding [8].

Drugs such as albendazole are often administered postoperatively to prevent recurrence in cases of spontaneous or iatrogenic rupture.

In rare cases of small or multiple cysts, medical therapy alone may be considered, although it is less effective for cysts larger than 10 cm.

We report a case of a complicated pulmonary hydatid cyst in a child who presented with vomica and respiratory distress.

## 2. Case Description

A previously healthy 11‐year‐old boy with no history of chronic illness, hospitalization, or prior surgery was admitted to the emergency department (ED) of a low‐volume pediatric center for sudden‐onset chest pain, shortness of breath, cough, and vomiting of clear, watery liquid (vomica). Symptoms began about 6 h before hospital admission. Demographic information indicated that the child lived in a rural area in Northern Italy, had owned a domestic dog for 4 years, and had never traveled outside his region.

Upon arrival at the ED, the child was tachypneic with expiratory wheezing. Vital signs showed hypotension (60/30 mmHg) and hypoxemia, with an oxygen saturation of 89% on room air.

After resuscitation with adrenaline, salbutamol, oxygen via nasal cannula, and intravenous (IV) fluids, the patient was hemodynamically stable and underwent laboratory testing and diagnostic imaging. Laboratory workup on arrival showed an elevated white blood cell count (17 × 10^3^/μL), with 81.2% neutrophils, 3.9% lymphocytes, and 9.6% eosinophils, and a C‐reactive protein (CRP) of 59 mg/L.

**FIGURE 1:**
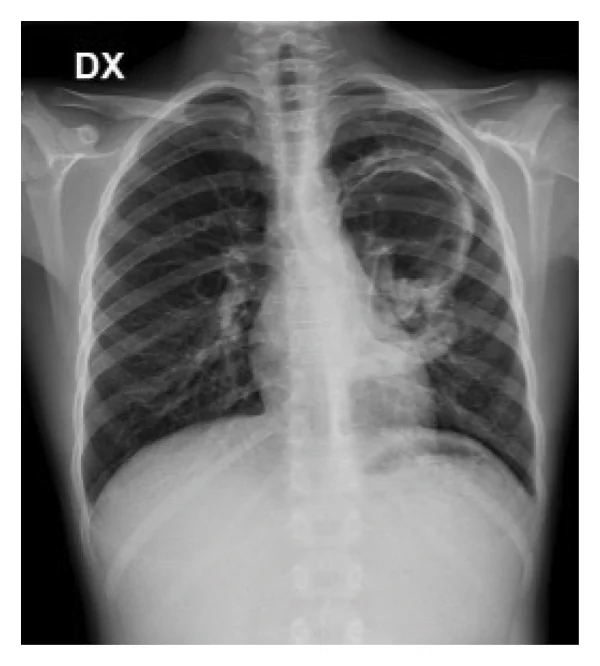
The chest X‐rays showed a large cyst in the upper left lobe of the lung, with the characteristic “water lily” sign, suggestive of the possible cyst rupture into the bronchial tree, and retention of the detached endocyst membrane.

A chest X‐ray revealed a large cavitary lesion measuring 8 cm in diameter in the upper left lung lobe, with atelectasis of the adjacent pulmonary parenchyma (Figure 1). The clinical presentation and radiologic features were suggestive of a hydatid cyst. Accordingly, a chest CT scan was performed to further characterize the lesion and to exclude other potential differential diagnoses, including lung abscess, congenital cystic malformation, and malignant lesions.

**FIGURE 2:**
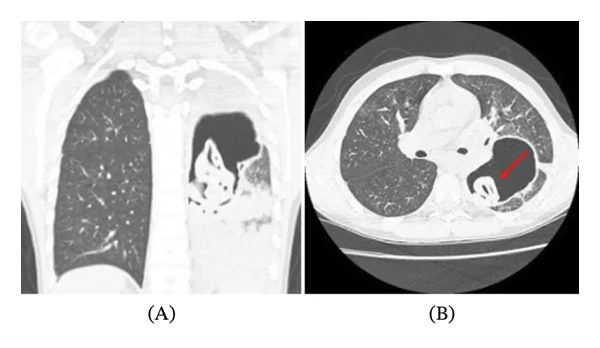
Preoperatory chest CT scan confirming the presence of a large cyst in the left upper lung lobe, measuring 7 × 6 × 9.5 cm, with bronchiectasis and “floating” membranes (the detached membrane) within the cyst cavity (red arrow) and signs of superinfection of the cyst.

CT scan confirmed a large cyst in the left upper lung lobe, measuring 7 × 6 × 9.5 cm, in communication with a bronchus image and “floating” membranes within the cyst cavity (“water lily sign”) indicating detachment of the endocyst from the pericyst, associated with signs of superinfection of the cyst, including ring enhancement, air bubble sign, and air–fluid level (Figures 2A and B). In addition, in the left lower lobe, there were areas of atelectasis and consolidation, indicating an associated secondary pulmonary infection, along with pleural thickening.

These findings were highly suggestive of a complicated hydatid cyst. The patient was therefore transferred to the infectious disease department of the regional pediatric referral center.

*Echinococcus granulosus* infection was further confirmed by a positive IgG ELISA test (titer 1:1280; normal values < 1:160).

Additional diagnostic work‐up, including abdominal ultrasound, brain MRI, echocardiography, and eye examination, ruled out other parasitic localizations.

**FIGURE 3:**
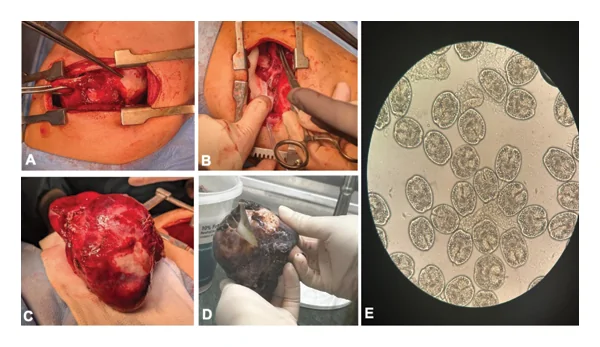
Hydatid cyst resected en bloc with the upper lung lobe (A, B, C, D); viable scolices visible on microscopy (E).

Preoperative medical treatment consisted of IV antibiotic therapy with cefotaxime (100 mg/kg) and teicoplanin (10 mg/kg), along with albendazole 400 mg twice daily for 14 days. Elective surgery was performed 2 weeks after hospital admission, when inflammatory indices had normalized (WBC: 5 × 10^3^/μL and CRP: 2 mg/L), secondary pneumonia was resolving, and the general condition had improved considerably. The procedure consisted of closure of the bronchial fistula, “en bloc” cyst removal, including the pericystic membrane, and two wedge resections of the adjacent fibronecrotic lung parenchyma via thoracotomy (Figure 3). Due to extensive adhesions of the pericyst and to reduce the risk of further dissemination of the cyst content, simple cystectomy and capitonage were not considered.

Histopathology of the specimen revealed lung parenchyma with a chronic inflammatory process, partially lymphocytic, with diffuse follicular aggregates and peribronchiolar and macrophagic granulomatous lesions, including giant cells, with blood congestion and hemorrhagic extravasations, marked eosinophilic component, and features of endoalveolar organization. A voluminous cystic formation was present within the parenchyma, with amorphous and necrotic content with lamellate membranes, calcifications, and structures referential to proctoscoliosis (Figure 3).

After surgery, the patient was admitted to the intensive care unit (ICU) for 48 h for close monitoring and was extubated on the first postoperative day (POD). Additional antibiotic therapy with piperacillin–tazobactam was administered for 10 days. Postoperatively, albendazole was administered for 2 weeks. The initial postoperative course was uneventful. During the hospitalization, the child maintained good general condition and did not experience dyspnea or require oxygen support. The chest drain was removed on POD 6. A chest X‐ray performed before discharge demonstrated a 6‐cm pneumatocele. The patient was asymptomatic and was discharged on POD 8. He was followed with a series of chest X‐rays in an outpatient setting.

One month after surgery, the child was readmitted to the hospital for infection of the residual pneumatocele.

The child was treated with IV 3rd‐generation cephalosporin and clindamycin for 1 week, followed by 7 days of oral antibiotic therapy with amoxicillin/clavulanate.

**FIGURE 4:**
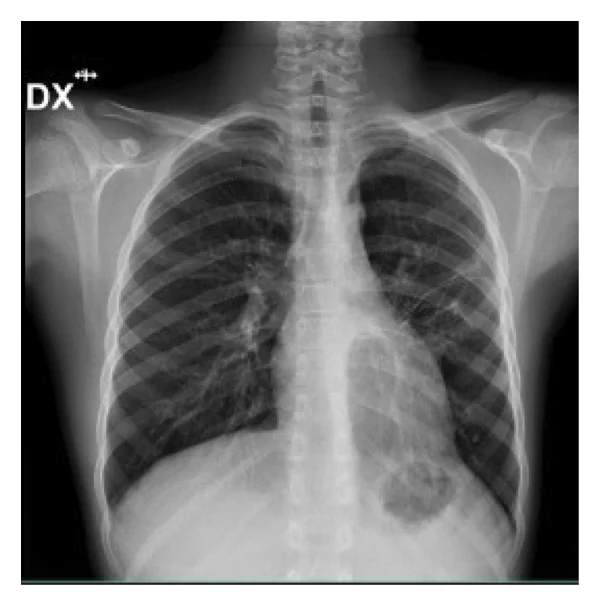
Complete resolution of the residual pneumatocele and re‐expansion of the lung at 1‐year follow‐up.

Complete resolution of the pneumatocele was achieved 8 months after surgery (Figure 4).

## 3. Discussion

Echinococcosis is a zoonotic disease for which epidemiological history is essential for diagnostic guidance. The clinician should consider hydatid disease if the child lives in or has traveled to endemic areas and has had contact with dogs or livestock.

The infection takes a long time to form hydatid larval cysts, most often in the liver in adults and in the lungs in children [3–6].

The diagnosis of pulmonary echinococcosis relies mainly on imaging, while clinical history and serological tests provide supportive information. Many cysts are detected incidentally in asymptomatic individuals.

Owing to the high elasticity of the pediatric lung, children often remain asymptomatic until a cyst reaches a considerable size. Cysts larger than 10 cm are typically defined as “giant” and are more frequently observed in pediatric patients, representing a distinct entity due to their higher risk of complications and morbidity [9]. When symptoms develop, they are usually related to the cyst’s mass effect on surrounding structures, with cough, chest pain, and dyspnea being the most common clinical manifestations.

In the present case, the patient remained completely asymptomatic until 6 hours prior to admission, when he developed acute respiratory distress and anaphylaxis following spontaneous rupture of the cyst. The measured maximum diameter of the cyst was 9.5 cm, which does not meet the conventional threshold for classification as a giant cyst. However, previous studies comparing radiologic features of complicated and uncomplicated cysts have demonstrated that ruptured cysts tend to have significantly smaller measured diameters than intact ones [10–12]. This reduction in size is likely attributable to partial emptying of the cyst contents after rupture.

Therefore, although the recorded diameter in our case falls below the established cutoff, it likely underestimates the cyst’s original size prior to rupture. Based on this consideration, the lesion can reasonably be regarded as a giant cyst in its prerupture state.

Undoubtedly, imaging is the most reliable method for detecting and characterizing hydatid cysts in the lungs [13]. The imaging characteristics of hydatid cysts depend on the stage of the disease.

A chest X‐ray is often the first test performed, and intact cysts appear as well‐defined, round lesions that may pose a differential diagnosis with congenital cystic malformations, tumors, lung abscess, and aspergilloma. More specific signs may appear, such as the “water lily sign,” in a ruptured cyst [14], as in our case. Infected cysts may be misdiagnosed as a lung abscess [15].

A CT scan is considered the gold standard for determining the size, number, and exact location of cysts, as well as for identifying complications such as ruptures or secondary infections.

Ultrasound is useful for evaluating peripheral lesions and is essential for screening the liver, as approximately 10%–30% of patients with pulmonary cysts also have liver involvement.

Laboratory tests and serology are considered complementary, as their sensitivity in purely pulmonary cases is lower (50%–60%) than in hepatic cases.

Serology (ELISA/Immunoblot) is used to detect IgG antibodies specific to *Echinococcus granulosus*, but a negative result does not rule out the disease. The IgG ELISA test is commonly used as a first screening test; if this is positive or equivocal, a more specific confirmatory test, the Western blot, is performed [16].

In this case, the IgG ELISA test, combined with the clinical history and the imaging results, was considered accurate for a definitive diagnosis.

Complete blood count may show eosinophilia, but this is present in only 15%–25% of cases [17].

Although the diagnosis was initially unexpected, given that Northern Italy is a nonendemic region, the acute clinical presentation, characterized by vomica and respiratory distress, was suggestive of a complicated hydatid cyst and facilitated prompt suspicion and rapid identification. In our case, the vomica did not completely eliminate the cyst contents, which can occur in rare cases of cyst rupture in the bronchus [18]. Ruptured cysts can cause severe anaphylactic reactions or later development of new cysts in the contaminated tissue and are associated with significant morbidity (up to 25% of cases) and a mortality rate that rarely exceeds approximately 1.7% of patients [19].

Bronchial and pleural ruptures often predispose to lung infection because solid remnants of the collapsed membrane in the cavity serve as a source of infection, often requiring prolonged antibiotic treatment and hospitalization [17]. In our case, the patients required 2 weeks of preoperative antibiotic treatment and 1 week of postoperative antibiotic treatment. Subsequently, a postoperative persistent residual cavity complicated by infection required hospital readmission and further antibiotic treatment.

As an increased mortality rate is associated with a prolonged interval between cyst rupture and surgery, some authors advocate that early surgical intervention as soon as the patient is in stable condition is mandatory [20].

However, cyst excision should be performed in an elective setting, possibly after a course of medical treatment with anthelmintics and antibiotics, to reduce inflammation and the risk of recurrence.

In our case, the interval between cyst rupture and surgery was 2 weeks, intended to reduce tissue inflammation. An earlier intervention in an infected lung would have required a more extensive resection and increased the risk of postoperative complications. Parenchymal‐sparing procedures are recommended over resection in pediatric patients and in endemic areas, where the risk of reinfection is higher. Indications for pneumonectomy and lobectomy remain controversial, and the resection rate ranged from 0% to 25%. Some authors recommend resection for giant cysts occupying more than 50% of a pulmonary lobe, multiple cysts, or when the surrounding lung tissue is completely destroyed [3, 4, 8, 15, 21, 22], while others perform cystectomy and capitonage regardless of cyst size or complications [23–25]. Postsurgical complications are not infrequent and include bronchopleural infection (often a consequence of cyst rupture and communication between the bronchial tree and the cyst cavity), fistulas, pneumothorax, atelectasis, and diaphragmatic paresis.

Complications are more common in complicated cysts, with air leak being the most common (7.9%) [8, 25].

Another possible postoperative complication is the passage of a fragment of the cyst membrane during operative manipulation into the airway, resulting in acute obstruction of the bronchus and respiratory failure. In this case, the postoperative course was complicated by an infected pneumatocele requiring serial follow‐up imaging and antibiotic therapy.

The recurrence of echinococcal cysts is rare and has been observed in cases in which hydatid cyst contents were spilled during surgery [13, 14, 16].

## 4. Conclusions

Lung echinococcosis is a zoonotic disease that is not infrequent in children, particularly in endemic areas.

Due to migratory flows, this infection could also be observed in countries not accustomed to such pathologies. Differential diagnosis should be considered when treating pulmonary masses or pneumonia, but vomica and respiratory symptoms lead the clinicians toward a complicated hydatid cyst.

## Funding

This research received no specific grant from any funding agency in the public, commercial, or not‐for‐profit sectors. Open access publishing facilitated by Universita degli Studi di Torino, as part of the Wiley ‐ CRUI‐CARE agreement.

## Consent

Written informed consent was obtained from the patient’s parents for the publication of this case report and any accompanying images. A copy of the written consent is available for review.

## Conflicts of Interest

The authors declare no conflicts of interest.

## Data Availability

Data sharing is not applicable to this article as no datasets were generated or analyzed during the current study.
